# Congenital limbal dermoid in a 10-year-old male

**DOI:** 10.11604/pamj.2023.44.30.38419

**Published:** 2023-01-17

**Authors:** Chrisann Saldanha, Sachin Daigavane

**Affiliations:** 1 Department of Ophthalmology, Jawaharlal Nehru Medical College, Datta Meghe Institute of Higher education and Research, Sawangi (Meghe), Wardha, Maharashtra, India

**Keywords:** Limbal dermoid, choristomas, astigmatism

## Image in medicine

The most typical choristomas, sometimes referred to as congenital benign tumors, are limbal dermoids. Limbal dermoids are typically seen in the inferotemporal quadrant, but they can sometimes exclusively be found in the conjunctiva and sclera or present fully within the perilimbal cornea. Despite being benign, progressive limbal dermoids may result in anisometropic amblyopia because of the development of corneal astigmatism, encroachment on the visual axis, and corneal penetration of fatty components. Anatomically, there are three different classes of limbal dermoids. Grade I limbal dermoids are small, superficial tumor lesions that are currently being treated with conservative medicine. Large Grade II limbal dermoids penetrate both Descemet's membrane and the corneal stroma. Grade III limbal dermoids impact all histological structures between the eyeball's anterior surface and the iris's pigmented epithelium, penetrate the anterior chamber and completely cover the cornea. The histologically abnormal tissues that can be found in limbal dermoids include epidermal appendages, connective tissues, skin, fat, sweat glands, lacrimal glands, neurological tissues, and hyperplasia. Here we represent a 10-year-old male with progressive limbal dermoid in the inferior quadrant in the left eye at 6 o'clock with hair follicles over it associated with the preauricular tag. A dermoid cyst was also present at 5 o'clock. It was progressive in nature and was associated with diminution of vision. Keeping that in mind surgical excision of the limbal dermoid was planned. The best timing and methods for surgical procedures in newborns and young children depend on the tumor size, tumor growth rate, and the affected locations.

**Figure 1:**
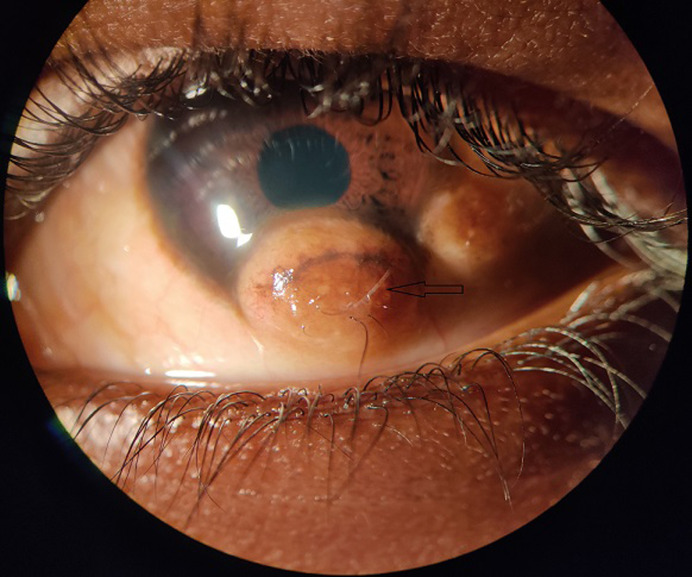
congenital limbal dermoid in a 10-year-old male

